# Effect of Ivabradine on Endothelial Function in Diastolic and Right Heart Failure Patients

**DOI:** 10.1155/2013/603913

**Published:** 2013-10-03

**Authors:** Arturo Orea-Tejeda, Karla Balderas-Muñoz, Lilia Castillo-Martínez, Oscar Infante-Vázquez, Raúl Martínez Memije, Candace Keirns-Davis, Joel Dorantes-García, René Narváez-David, Zuilma Vázquez-Ortíz

**Affiliations:** ^1^Heart Failure Clinic, Instituto Nacional de Ciencias Médicas y Nutrición Salvador Zubirán, Mexico; ^2^Instrumentation Department, Instituto Nacional de Cardiología “ICh”, Mexico; ^3^Massachusetts General Hospital, Boston, MA, USA; ^4^Cardiology Department, Instituto Nacional de Ciencias Médicas y Nutrición Salvador Zubirán, Mexico

## Abstract

*Background*. Ivabradine is an If ion current inhibitor that has proved to reduce mortality in patients with systolic heart failure by slowing heart rate without decreasing myocardial contractility. Photoplethysmography is a simple, low-cost optical technique that can evaluate vascular function and detect changes in blood flow, pulse, and swelling of tissular microvascular space. *Objective*. To evaluate the effect of ivabradine on endothelial function by photoplethysmography in diastolic and right heart failure patients. *Methodology*. 15 patients were included (mean age of 78.1 ± 9.2 years) with optimally treated diastolic and right heart failure. They underwent photoplethysmography before and after induced ischemia to evaluate the wave blood flow on the finger, using the maximum amplitude time/total time (MAT/TT) index. Two measurements were made before and after oral Ivabradine (mean 12.5 mg a day during 6 months of followup). *Results*. In the study group, the MAT/TT index was 29.1 ± 2.2 versus 24.3 ± 3.2 (*P* = 0.05) in basal recording and 30.4 ± 2.1 versus 23.3 ± 2.9 (*P* = 0.002), before versus after ischemia and before versus after Ivabradine intervention, respectively. *Conclusions*. Ivabradine administration improves endothelial function (shear stress) in diastolic and right heart failure patients.

## 1. Background

Diastolic dysfunction has been associated with symptoms of congestive heart failure in patients with preserved left ventricular ejection fraction [1]. In the largest single-center study with approximately 36, 000 outpatients with normal LVEF, some authors have shown that diastolic dysfunction is an independent predictor of all-cause mortality [2].

In outpatients with heart failure with preserved ejection fraction (HFpEF) at the baseline echocardiogram, worsening of diastolic function in a follow-up study is also an independent predictor of all-cause mortality [3]. In addition, Achong showed that improvement in diastolic function was associated with increased survival (*P* = 0.05) in a mixed cohort of inpatients and outpatients with normal or mild systolic dysfunction [4], while the more advanced the stage is, the higher the filling pressures and the worse the outcomes are [5].

In patients with atrial fibrillation, if diastolic function was assessed, it was based on deceleration time of mitral E-wave velocity and tissue Doppler imaging (i.e., peak early mitral inflow velocity/diastolic early tissue velocity [E/e′]) [6].

Ivabradine added to recommended treatment, improved the outcome of heart failure patients reducing cardiovascular death and hospitalizations rate [7]. In patients with coronary disease and left ventricular dysfunction also the benefit was observed [8]. In experimental studies, ivabradine have been demonstrated that reduces fibrosis and improve endothelial function [9–12].

Ivabradine also has demonstrated a favorable effect on LV remodeling after 8 months of followup as well as and antianginal effect [13]. 

 Hyperemic coronary blood flow velocity also increases after ivabradine treatment, probably because the diastolic period is prolonged (per cardiac beat and per minute). It has been speculated that the most probable explanation of the improvement of ventricular relaxation caused by ivabradine treatment could be its effect on coronary blood flow velocity during hyperemia [14]. 

To evaluate the effect of ivabradine on endothelial function by photoplethysmography in patients with right heart failure and preserved ejection fraction, we performed this open-label clinical trial.

## 2. Methods

### 2.1. Study Population

 This open-label clinical trial included ambulatory patients who came to the Heart Failure Clinic of the Instituto Nacional de Ciencias Médicas y Nutrición “Salvador Zubirán.” Patients were recruited if they were men or nongravid women with more than 18 years of age with a confirmed diagnosis of stable heart failure with preserved ejection fraction in New York Heart Association functional classes II to III. Candidates were excluded if they had had myocardial infarction, unstable angina or a history of myocardial revascularization (percutaneous transluminal coronary angioplasty or aortocoronary bypass grafts), cerebrovascular events during the previous 3 months, dysfunctional prosthetic heart valve, obstructive or nonobstructive cardiomyopathy, uncorrected congenital heart disease, active myocarditis, a history of resuscitation from sudden death, or severe arrhythmias.

Heart failure was established by signs and symptoms as well as echocardiographic and radioisotopic ventriculography findings. Preserved ejection fraction was defined as a left ventricular ejection fraction ≥ 50%, LVEDVI < 97 mL/m^2^, left atrial diameter > 40 mL/m^2^, tissue doppler E/E′ > 15 echo-blood flow Doppler E/A in >50 years <0.5, and DT in >50 years >280 ms [15]. Right ventricular dysfunction was defined as ejection fraction ≤ 35% measured by radioisotopic ventriculography [16, 17].

 All patients received standard heart failure therapy and their comorbidities (diuretics, angiotensin-converting enzyme inhibitors, angiotensin II antagonists, aldosterone receptor blockers, digitalis, and beta-adrenoreceptor blockers), at their maximum doses tolerated. Some patients that developed atrial fibrillation also received digital.

### 2.2. Study Design

This was an investigator-initiated, single center, single-arm, open-label clinical trial.

After baseline measurements, in addition to conventional therapy, patients received an average of ivabradine 12.5 mg (10–15 mg) a day, according their tolerance during 6 months of follow-up [18, 19]. Patients underwent 2D and Doppler echocardiograms and radioisotopic (rest/effort) left and right ventriculography before and after oral ivabradine. 

#### 2.2.1. Photoplethysmography

 A baseline digital photoplethysmographic wave was recorded for 30 seconds. The forearm was then compressed with a sphygmomanometer cuff for 5 minutes using a pressure of 30 mmHg above the systolic arterial pressure recorded (ischemic phase). The compression was then released and the digital photoplethysmographic wave was recorded for 120 seconds. The wave was analyzed at 30-second intervals for comparison with the baseline values. The most representative waves were selected from the recording of each interval, and the maximum amplitude time (MAT) and total time (TT) were measured in order to calculate the MAT/TT index. A MAT/TT index of less than 30 was considered normal, as proposed in other studies [20, 21].

Cardiologists who performed the echocardiograms and radioventriculography did not have access to patients' information.

### 2.3. Statistical Analysis

 Continuous variables were expressed as mean ± standard deviation (SD) and categorical variables as percentages. To compare the changes from baseline to 6 months, a paired *t*-test was used. A *P* value of <0.05 was considered statistically significant. All analyses were performed using a commercially available package (SPSS for Windows, version 17.0 SPSS Inc.).

## 3. Results

Fifteen patients (73.6% female) were studied. Arterial hypertension and hypothyroidism (under treatment and well controlled) were the most common comorbidities with patients in functional classes (NYHA) II and III (Table 1). It is important to note that COPD and ESKD patients were not excluded from the study. Concomitant medication was as follows: diuretic (73%) and adrenergic beta blocker receptor (BB, 73%) agents were the most commonly employed; 46.6% also received mineralocorticoid receptor antagonists (MRAmedications) and angiotensin-converting-enzyme inhibitors (ACEIs)/angiotensin receptors blockers (ARB).

 Patients received an average of 12.5 (range 10–15) mg/day during the 6 months of followup. It was particularly interesting that heart rate did not decrease in any patients below the 10% recommended (88 versus 82 beats/min) in the literature in spite of the top doses received.

**Figure 1:**
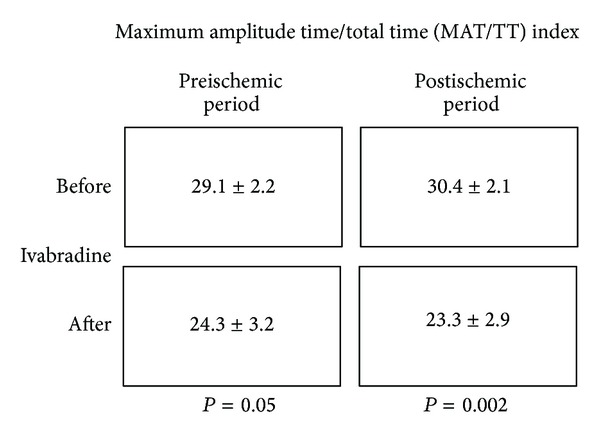
Pre and after ischemic period, before and after 6 months of follow-up of oral ivabradine.

Figure 1 shows the maximum amplitude time/total time (MAT/TT) index before and after the followup. A significant increase in pre- and post-ischemic periods after ivabradine administration is evident when basal values are compared with those at the end of followup.

 Improvement of the endothelial-dependent vasodilatation expressed by significant changes observed in the photoplethysmographic curves occurred concurrently. All patients had some degree of clinical improvement, 8/9 (88.8%) from NYHA III to II and 4/6 (66.6%) from II to I, respectively, although this did not achieve statistical significance (*P* = 0.08).

 With respect to cardiac structural changes, in the echocardiographic study, only right ventricular diastolic diameter (40.5 ± 7.8 versus 36.4 ± 5.3; *P* = 0.05) was significantly different after the followup. There was also a reduction of 8.15% in the systolic pulmonary arterial pressure (59.6 ± 8.4 versus 54.9 ± 10.2;  *P* = 0.05).

## 4. Discussion 

 Approximately 50% of patients with heart failure (HF) have normal or preserved left ventricular ejection fraction (HFPEF) [22], and their prognosis is similar to that of patients with HF with reduced LVEF (HFREF) [22, 23]. Left ventricular diastolic dysfunction (LVDD) plays an important role in patients with HFPEF [24] and could be due to structural and molecular abnormalities of the cardiovascular system. These abnormalities include myocardial ischemia, cardiac hypertrophy, cardiac inflammation [25], and ventricular vascular stiffening, in part due to the reduced effects of nitric oxide and impaired endothelial function [26]. Borlaug et al. recently demonstrated that global cardiovascular reserve functions, including endothelial function, are impaired in subjects with HFNEF who have hypertension, a very frequent cause of HF [27]. In HFREF, coronary endothelial function is also impaired [28], and it has been reported that peripheral endothelial dysfunction is associated with the severity of HF symptoms and clinical outcome in patients with HFREF [29, 30]. Moreover, vascular stiffness and resistance with elevated blood pressure has been proposed as a potential important noncardiac factor in patients with HFpEF [31].

Endothelial dysfunction has been shown to be involved in the pathogenesis of HF, mainly HFREF. Several studies have reported that peripheral endothelial dysfunction is associated with the clinical outcome in patients with HFREF [30]. Borlaug et al. recently reported that subjects with HFpEF had limited arterial vasodilator response to exercise, which might impair cardiac output reserve under stress [32].

Peripheral endothelial function is impaired in patients with HFpEF, and when it is evaluated as a reactive hyperemia by peripheral arterial tonometry (RH-PAT), it significantly correlates with future cardiovascular events. Peripheral endothelial function is thus an independent predictor after adjusting various clinical parameters [33]. Indeed, the prognostic impact of the reactive hyperemia index in patients with HFpEF suggests that endothelial dysfunction may not be a passive finding, but may rather play an active and important pathophysiologic role in HFpEF [32]. When matched in patients and controls for diabetes and hypertension, more endothelial dysfunction was found in patients with HFPEF, who were notably more obese than controls [34].

 The results of the systolic heart failure treatment with the If inhibitor ivabradine trial (SHIFT) showed that treatment with ivabradine added to conventional therapy for HF was associated with an 18% reduction in the relative risk for the primary composite endpoint of cardiovascular death or hospitalization for worsening HF (*P* < 0.0001) [7]. It also had a positive effect on LV remodelling in the echocardiographic substudy of the BEAUTIFUL (morBidity-mortality EvAlUaTion of the If inhibitor ivabradine in patients with coronary disease and left ventricULar dysfunction) study [8].

 In experimental studies, ivabradine has been demonstrated to reduces fibrosis and improve endothelial function [9–12], together with its antiischemic and antianginal effects [13] which could explain why all our patients improved their functional class, possibly associated with increased hyperemic coronary blood flow velocity. It may also be explained by the prolonged diastolic period (per cardiac beat and per minute). Moreover, we can speculate that the endothelial-dependent vasodilatation expressed by the significant changes observed in the photoplethysmographic curves in peripheral and coronary territories plays an important role in improving coronary blood reserve as has already been described [35, 36], and flow velocity may have a direct effect on coronary vessels and reduced ventricular wall tension, with improvement of ventricular relaxation [14], added to diminished right ventricular diastolic diameter with significant reduction of arterial pulmonary pressure, which probably reflects improved coronary perfusion pressure (aortic mean pressure/coronary sinus ratio).

In experimental postinfarction settings, both cardiac [37] and pulmonary vascular [38] endothelial dysfunction may contribute to the development of heart failure through endocardial and myocardial capillary endothelial abnormalities [39] and could explain the impaired left ventricular relaxation in pressure-overload hypertrophy [40].

Preserved ejection fraction is present in almost 50% of heart failure patients and is cause of half of HF hospitalizations, and traditional HF treatment is not effective. A recently published pathophysiology-based novel pharmacotherapy for these patients considers spironolactone, aliskiren, and neprilisyn as therapeutic options for HFPEF because of their anti-hypertrophic and anti-fibrotic effects [41]. Combined ventricular and vascular stiffening involving both the systemic and pulmonary circulations, plays a role in the pathophysiology of HFpEF [42, 43]. Thus, the effects of ivabradine on endothelial function, that we observed, may represent a major advantage when it is used to treat left diastolic dysfunction because of its secondary impact on pulmonary arterial hypertension and damaged right ventricular function.

## 5. Limitations

The number of patients studied was small, and the intervention period was short. It is probable that a longer followup would show changes in variables such as left ventricular diastolic diameter and other structural characteristics. In addition, the lack of direct quantification of pulmonary pressures is a drawback. 

 Our findings support continued investigation into the effects of ivabradine on right ventricular function, systemic arterial pressure, and systolic pulmonary arterial pressure in heart failure patients with preserved ejection fraction. More studies are required to evaluate the effects observed on a larger number of patients for a longer period.

## Figures and Tables

**Table 1:** Demographic and clinical characteristics of the CHF patients.

Variables	*N* = 15
Age (years)	78.1 ± 9.2
Female (%)	73.3
Arterial hypertension (%)	73.3
Hypothyroidism (%)	53.3
Diabetes mellitus (%)	33.3
COPD (%)	33.3
Dyslipidemia (%)	26.6
End Stage Kidney disease (%)	26.6
Functional class (NYHA):	
II	60
III	40
